# Rural veteran perception of healthcare access in South Carolina and Florida: a qualitative study

**DOI:** 10.1186/s12913-024-11241-3

**Published:** 2024-07-20

**Authors:** Maria Mercedes Rossi, Heidi L. Radunovich, Michelle A. Parisi

**Affiliations:** 1 Department of Food, Nutrition, and Packaging Sciences C228 Pool Agricultural Center, Clemson University, Clemson, SC-29631 USA; 2 Department of Family, Youth, and Community Sciences 3008-B McCarty D, University of Florida, Gainesville, FL-32611 USA; 3 Department Nutritional Sciences 206 Hoke Smith Annex, University of Georgia, Athens, GA-30602 USA

**Keywords:** Rural Veterans, Families, Healthcare access, Qualitative study

## Abstract

**Background:**

Access to mental and physical healthcare in rural areas is challenging for Veterans and their families but essential for good health. Even though recent research has revealed some of the challenges rural Veterans face accessing healthcare, a complete understanding of the gap in access is still unclear.

**Methods:**

This qualitative study aimed to explore participants’ perceptions of healthcare access. Structured interviews were conducted with 124 Veterans and spouses of Veterans from rural qualifying counties in South Carolina and Florida.

**Results:**

The study’s results revealed five main dimensions of access: geographic proximity, transportation, communication, cultural competence, and resources. Distance to service needed can negatively impact access for Veterans and their families in general, especially for those whose health is declining or who cannot drive because of their age. Lack of transportation, problems with transportation services, and lack of public transportation can lead to delays in care. Additionally, the lack of communication with the Veterans Affairs (VA) Health System and with the healthcare team, as well as inefficient communication among the healthcare team, lack of coordination of care between the VA health system and community providers, and the lack of cultural competence of healthcare providers and contracted personnel made access to services even more challenging.

**Conclusions:**

Improving communication can help to develop a sense of trust between Veterans and the VA, and between Veterans and spouses with the healthcare team. It can also lead to increased patient satisfaction. Ensuring healthcare providers and contracted personnel are culturally competent to talk and treat Veterans can improve patient trust and adherence to treatment. Lastly, resource-related challenges included financial problems, lack of prompt access to appointments, lack of providers, limited access to local clinics and hospitals, limited local programs available, and reimbursement issues.

**Supplementary Information:**

The online version contains supplementary material available at 10.1186/s12913-024-11241-3.

## Background

While access to healthcare is essential for good health, many U.S. military Veterans and their families living in rural areas report not having access to necessary healthcare in their communities. The Institute of Medicine Committee on Monitoring Access to Personal Healthcare developed a set of indicators to measure problems in accessing specific healthcare services. They defined access as “the use of personal health services to achieve the best health outcomes.” [1] Different factors, such as geography, distance, finances, and culture, can affect Veterans’ healthcare access [2]. Inaccessible healthcare because of geographical location, for instance, means that Veterans must travel long distances to get healthcare [3]. Distance to service can negatively affect those with limited health function and financial resources, those who need routine specialty care diagnostics, and those in emergencies [4]. The cultural access dimension involves differences in expectations and communication styles between healthcare providers and Veterans [2]. Cultural differences might be related to race and ethnicity, socioeconomic status, age, gender, religion, or other factors [3]. Research shows that, on occasion, there is cultural indifference from healthcare providers to service members or Veterans of color who may have more adverse physical and mental health conditions than their White counterparts [5]. Research also shows that healthcare providers’ lack of knowledge about military culture can create a barrier between themselves and the Veteran patient, affecting communication and direct care [6]. For instance, Veterans are at risk of health disparities regarding their mental health, including post-traumatic stress disorder (PTSD), military sexual trauma (MST), and substance use, as mental health providers need to fully understand those mental health issues to provide best practices and treatment options for this population [7]. This knowledge gap about Veterans negatively affects these patients, often causing them to drop out of care, be misdiagnosed or lose trust in the healthcare provider’s treatment plan, or they may seek care only when the disease is in an advanced stage [8]. Financial access refers to the ability to pay healthcare expenses such as copayments, lack of adequate coverage, and income ineligibility for government assistance [2, 3]. Another dimension is timeliness or delay in receiving services [2]. Even though appointments might be geographically available, affordable, and culturally competent, long waiting times for visits and delays in treatments have also been identified as significant barriers to healthcare for Veterans [2, 7].

In 2006, the Veteran Health Administration Office of Rural Health (VHA-ORH) was established to support the needs of Veterans enrolled in the Veterans Affairs (VA) healthcare system and reduce rural healthcare disparities [8]. Approximately 2.8 million enrolled rural Veterans rely on the Veterans Affairs (VA) healthcare system. Over one-half of those rural Veterans enrolled in the VA health system (56.0%) are over the age of 65. This elderly population is more likely to have complex medical conditions such as diabetes, high blood pressure, and heart conditions. The next generation of rural Veterans will also need access to care because they are more likely to have multiple health conditions [9].

Over 400,000 Veterans live in South Carolina (SC), which represents 2% of the Veteran population in the U.S. (*n* = 19,928,795) [10]. SC ranks 18th in the nation for the number of Veterans residing in the state [11]. Of those Veterans, 125,318 live in rural counties [12]. SC is ranked 8th in the country for the number of military retirees, with a population of 397,649 Veteran retirees [13]. Close to one-half of the SC Veteran population (45.11%) is over 65 (*n* = 181,617). On the other hand, Florida (FL) ranks 3rd in the nation for the number of Veterans, with a Veteran population of 1,542,770, representing 8% of the total U.S. Veteran population. Of those, 167,816 live in rural areas. Additionally, over one-half of the Veteran population in FL (50.6%, *n* = 780,845) is over 65, and 13% of the Veterans are retirees [14].

Rural Veterans tend to have lower physical health-related quality of life scores than those in urban or suburban areas [15]. They also have more physical health co-morbidities than their counterparts [15, 16]. Providing equal access to services has been a top priority for the U.S. Veteran health system; however, access in rural areas is still a challenge due to the limited number of specialized providers, limited options for assessment and treatment referrals, lack of providers, and provider’s lack of cultural awareness of the community [5]. Research revealed a gap in knowledge about working and treating the military and Veteran population who are affected by service-related mental health problems and the urgency to incorporate military cultural competency in treatment plans to address the unique dynamics of this population [6, 7]. Recent research also has revealed some improvements the VA Veterans Health Administration (VHA) Office of Rural Health (ORH) made to help close the gap in access [17]. Initiatives include developing partnerships with community health centers, rural health clinics, and mobile clinics, delivering care in person or through telehealth [18]. Even though the Department of Veterans Affairs is the largest government organization that offers services for the mental and physical healthcare needs of this population, roughly 1/3 (over 9 million) of the Veteran population is enrolled in the VA Healthcare System. Veterans who are employed often use their private health insurance benefits. Due to this situation, Veterans are being treated by civilian health professionals who need knowledge about how the military culture shapes their lives and experiences to provide culturally sensitive treatment and interventions to Veterans [7]. According to research, the use of concurrent VA and non-VA healthcare services is relatively high among rural Veterans. The estimated range of Veterans using dual or concurrent services is between 28% and 75%, particularly for those living far from the VA [19]. Due to a lack of communication and coordination of services between community institutions and the VA, Veterans face the challenge of coordinating their care [20]. This situation has led to poor patient outcomes, lack of continuity of care, or duplicated, delayed, and contradictory medical services [19, 20] which on occasion has led to patients being out of care [20].

This study aimed to gain a deeper understanding of the barriers and constraints that rural Veterans and their families face to accessing healthcare in SC and FL. This research was conducted in partnership with [University] and supported by a grant from the Veterans Rural Health Resource Center-Gainesville (VRHRC-GNV).

## Methodology

### Study design

From April to October 2022, a qualitative study was conducted to identify factors that affect how rural Veterans and their families access physical and mental healthcare in SC and FL. The University of Florida developed the study procedures and replicated them with minor variations for SC. The Clemson University Human Subjects Institutional Review Board (IRB) and the University of Florida Human Institute Review Board (IRB) approved each qualitative study.

### Sampling

We used a non-probability sampling strategy, a convenience sampling technique for participant recruitment, where participants were selected ad hoc. Participants have in common (homogeneous) their Veterans status or their spouses of Veterans status and their rurality; this is living in a rural county in South Carolina or Florida. To determine eligibility, we used the Rural-Urban Commuting Areas (RUCA) system to define rurality. RUCA considers population density and how closely a community is socio-economically connected to larger urban centers using positive whole numbers (1–10) to outline metropolitan, micropolitan, small-town, and rural commuting areas based on the size and direction of the primary and most prominent commuting flows. Counties were included if they had RUCA zip codes categorized as micropolitan (RUCA codes 4–6) or rural (RUCA codes 7–10) [21]. Twenty out of 46 counties in SC had a RUCA rating of four or higher. A county with a rating of four or higher is considered rural [22] and included Abbeville, Allendale, Bamberg, Barnwell, Cherokee Chesterfield, Clarendon, Colleton, Dillon, Georgetown, Greenwood, Hampton, Lee, McCormick, Marion, Marlboro, Newberry, Oconee, Orangeburg, Williamsburg. In FL, 23 of 67 counties had a RUCA rating of four or higher Bradford, Calhoun, Columbia, DeSoto, Dixie, Franklin, Glades, Hamilton, Hardee, Hendry, Holmes, Jackson, Lafayette, Levy, Liberty, Madison, Monroe, Okeechobee, Putnam, Suwanee, Taylor, Union, and Washington. Participants were Veteran adults and spouses living in any of the specified counties. Recruitment strategies included announcements through County Veterans Affairs Offices, County Extension offices, schools, supermarkets, a Veteran museum, Veterans Organizations, Facebook pages, advertisements in the newspaper, and attendance at in-person events for Veterans.

### Data collection

Data were collected in SC and FL. A total of 124 Veterans and spouses participated in a structured interview by connecting with researchers via their computers/ laptops, phones, or other electronic devices. Of those participants, 62 Veterans and 11 spouses were from SC, while 36 Veterans and 15 spouses were from FL.

The length of the interview ranged from 20 to 60 min. Participants verbally confirmed informed consent during the interview process. The audio recording transcriptions were automatically generated for each interview using a computer conferencing software. After the interviews, audio recordings were reviewed, and transcriptions were verified for accuracy [23]. After completing the interview, participants received an electronic or physical gift card worth $25 as a token of appreciation for their time.

### Instrumentation

The structured questionnaire was developed by the investigators for this study. The instrument tool was divided into three major sections: demographic information, physical and mental health needs and barriers, and knowledge and experiences with Cooperative Extension Services programming. Probes and follow-up questions were used to clarify and gather in-depth information.

### Data analysis and interpretation

ATLAS.ti Web (Mac version 22), a computer-assisted qualitative data analysis software, was used for data analysis. Following the Ryan and Bernard methodology, three interview transcripts were analyzed by manual coding to initiate the generation of the codes, themes, and sub-themes in the codebook [24]. Additional research team members practiced manual coding on ten interview transcripts without software, agreed upon coding characteristics, and modified the codebook for interrater consistency (in-vivo coding). The codebook was uploaded into ATLAS.ti software and used to categorize participant responses. Codes and respective definitions were refined and merged. To test for the reliability of the data, Krippendorff’s Cu α, a measure of intercoder agreement, was calculated for the semantic domains Healthcare (Cu α = 0.603) and Experience (Cu α = 0.958). Since SC and FL shared the same interview protocol, interviews from both states were combined and uploaded into Atlas.ti. The lead researcher from SC met with the principal investigator from FL to discuss and confirm the codes from the original codebook created for SC. Differences in coding were examined until a consensus was reached [25]. Saturation was reached as the researchers arrived at the point where no new information emerged during the coding.

To establish if data from Veterans who participated in our study represent the entire Veteran population, we isolated the Veteran demographic data and compared our Veteran population, Veteran national data, and Veterans residing in SC and FL. We analyzed demographic characteristics such as gender and age. These results showed similar profiles, indicating similarities among our Veteran population and allowing the possibility of generalizing results to the entire Veteran population.

## Results

### Demographic characteristics

Table 1 presents the demographic characteristics of our sampled population. A total of 124 participants completed the interview; 79% were Veterans, and 21% were spouses. Most were males (64%) and married (72%). Over one-half were over the age of 65 (51%), 37% ranged from 40 to 64 years, and 12% from 18 to 39 years. Less than one-half were retired (48%), and 6% were retired but still employed. About one-third (31%) were employed full-time. Approximately 47% reported an annual household income below $50,000, and 66% lived in a 2–3-person household. Regarding Veterans’ healthcare coverage, less than one-half of our participants (37%, *n* = 38) used non-VA healthcare coverage (private or Government). In contrast, 44% (*n* = 43) used a combination of non-VA healthcare coverage and the VA Healthcare System as coverage. The reasons for not utilizing the VA Healthcare System were as follows: 33% were told or believed they did not qualify for VA benefits, 18% used private insurance from work, and 12% because of a high distance to a VA Hospital/clinic to attend either to their primary care provider or specialty doctor. Only 18% had exclusively used the VA Healthcare System as coverage (*n* = 18). Close to one-quarter of our Veteran population (42%, *n* = 40) has a primary care provider (PCP) outside the VA, 27% through the VA (*n* = 26), and 4% did not specify whether the primary care provider is through the VA or non-VA (*n* = 4).

**Table 1:** Participants’ demographic characteristics

Characteristics	Frequency		Percentages	
*Participant type*				
Veteran	98		79%	
Spouse	26		21%	
*Gender*				
Female	45		36%	
Male	79		64%	
*Age Range*				
18–39 years	14		12%	
40–64 years	46		37%	
65 years +	64		51%	
*Race/Ethnicity*				
Non-Hispanic White	96		77%	
Non-Hispanic African American	20		16%	
Hispanic/ Latino	4		3%	
Mixed Race	3		2%	
*Marital Status*				
Divorced	12		10%	
Married	89		72%	
Partnered/ unmarried	2		2%	
Separated	1		1%	
Single/ Never Married	9		7%	
Widowed	11		9%	
*Annual Household Income*				
$24,999 or below	15		12%	
$25,000 to $44,999	17		14%	
$45,000 to $74,999	40		32%	
$75,000 to $99,999	18		15%	
$100,000 and above	26		21%	
Don’t know/Prefer not to answer	7		6%	
*Household size*				
1 person	24		19%	
2–3 people	82		66%	
More than 4 people	17		13%	
Does not apply (lives in a nursing home)	1		1%	
*Employment Status*				
Retired	59		48%	
Unemployed	15		12%	
Working full-time	39		31%	
Working part-time	3		2%	
Retired but working	8		6%	
*Veterans’ Healthcare Coverage*				
Use the VA healthcare only	18		18%	
Dual VA and non-VA healthcare	43		44%	
Use a non-VA healthcare	37		38%	
Total number of Veterans	**98**		**100%**	
*Veteran’s Use of the VA Community Care Program*				
Never used the VA Community Care Program	67		68%	
Used the VA Community Care Program	27		28%	
Questions not asked	4		4%	
Total number of Veterans	98		100%	
*Veteran’s Use of the VA or any Veteran Specific Health Resource*				
Never used the VA or any Veteran specific health resource	25		26%	
Use the VA or any Veteran specific health resource	73		74%	
Total number of Veterans	98			100%

On the contrary, 26% did not mention having a primary care provider (*n* = 25). Of those having dual coverage (*n* = 43), 28% chose a PCP outside the VA, 30% chose a PCP within the VA, and 7% did not specify if the PCP is with the VA healthcare system or non-VA. Conversely, 35% did not mention whether they have a PCP.

Veterans were asked if they had ever used VA healthcare or any VA health-specific resources. Most Veterans (74%, *n* = 73) responded that they used it. In contrast, 26% answered that they did not. Then, we asked Veterans if they had ever used a resource called the VA Community Care Program. Most participants (68%, *n* = 67) responded that they had never used or heard of it, while only 28% used it or had used it in the past (*n* = 27).

### Barriers to access to physical and mental healthcare

In response to the question, “Are you able to get the care you need? If not, what gets in your way?” the semantic domain barriers to healthcare access were identified and organized in the following dimensions: geographic proximity, transportation access, communication, cultural competence and resources. It is essential to highlight that our participants shared not only their own barriers to accessing healthcare but also the barriers their peers and or their family members encounter.

#### Geographic proximity dimension

Geographic proximity to the nearest facility or provider includes road distance and travel time. The perceived access is the travel time or number of miles reported by participants. Of 124 participants, 44% (*n* = 54) reported experiencing challenges in accessing healthcare due to a high travel distance or travel time to the needed service, either to their primary care provider (PCP) or specialty care provider (SCP). As a Veteran said, “*Because I stayed in rural towns, the problem is getting there (VA medical facility), the distance between where I am and where I can get the healthcare assistance*.” As a spouse also shared, *“I tried to look for counseling for myself and a marriage counseling. We went to the VA— my husband had a therapist there, and we went once. He told us to go to the Vet Center in Gainesville, and we tried that as well, but our baby was a little younger, and it was an hour’s drive each way. So we did not, you know, like, finish the treatment.”*

#### Transportation access dimension

Transportation was another main barrier identified by participants to access healthcare. Seventeen percent reported having a transportation problem. Lack of transportation to access services needed, lack of public transportation, inability to drive due to age or medical condition, and problems with transportation services were common problems. A Veteran shared with us, “*Some Veterans out in these rural counties, especially the older ones, can’t drive due to the age, and disabilities, so it just makes it really hard for them to get back and forth.”* Table 2 presents the descriptive statistics of participants sharing geographic proximity or transportation barriers.

**Table 2:** Geographic proximity and/or transportation access barriers

Barriers		Barrier	Frequencies
*Geographic Proximity Barrier*			
Experiencing any geographic proximity barrier		54	44%
Did not mention a geographic proximity barrier		70	56%
**Total number of participants**		**124**	**100%**
*Travel Distance*			
30 miles or less		3	9%
31–50 miles (one-way)		6	17%
51–79 miles (one-way)		7	20%
More than 80 miles (one-way)		3	9%
High Distance (without specifying the number of miles)		16	46%
*Travel Time*			
31–60 min (one-way)		8	40%
61–120 min (one-way)		5	26%
More than 2 h (one-way)		6	32%
*Transportation Barrier Access*			
Experiencing a transportation problem		21	17%
Did not mention a transportation problem		103	83%
**Total number of participants**		**124**	**100%**
*Type of Transportation Problem*			
Lack of transportation to access services		9	43%
Lack of public transportation		5	19%
Veteran unable to drive because of age or medical condition		4	24%
Lack or have problems with transportation services		3	14%*

Note: *The different types of transportation problems do not round out to 100% since a participant can be experiencing more than one type of barrier

#### Resources dimension

Lack of prompt access to appointments, financial problems, lack of providers, limited access to local clinics and hospitals, limited local programs available, and problems with reimbursement were the resource barriers identified by the participants. As a spouse shared, “*the biggest issue has been getting a timely appointments. He is a 65% disabled rating* now *now, and he’s had a lot of issues, trying to get timely appointments.*” A Veteran told us, *“It’s so hard to get into the VA to make an appointment. If you call today or send a message today to try to make an appointment, it will probably be almost a month before I can get in. I have just recently had some very, very, very bad pain. It was a bulging disc that got worse. I went to a civilian doctor and not the VA because they took too long, and I couldn’t function that way .”*

#### Cultural competence dimension

Cultural indifference based on race, age and gender, lack of tact/knowledge on how to talk to Veterans, lack of knowledge on how to treat Veterans and lack of knowledge about Veterans in general encompasses the lack of cultural competence barriers identified by Veterans and some spouses talking about their Veteran spouse/partner situation. Anecdotal comments from a spouse exemplify this dimension: “*My husband has several issues like PTSD, depression, and substance abuse. And it’s been hard to find somebody to help with that. He went to Bay Pines in the St. Petersburg area (non-VA clinic), but they don’t see anybody that doesn’t live there in the area. He also has chronic pain because he broke his back. And it seems like the doctors all want to do is prescribe him painkillers. But he has a substance abuse problem, so he’s not taking anything right now, prescribed.*” A veteran also shared, “*Those are the kinds of people who work at the VA that need to be removed, like that lady (referring to someone from administration at a VA Hospital). I am not only a Veteran but also worked for the Veterans Administration for 10 years. I worked with Veterans, and I worked with other people in that building. A lot of them are non-Veterans, and many don’t care, and or they don’t get it.”*

#### Communication dimension

Inefficient communication between participants and the healthcare team (including doctors, nurses, administrative and support staff, and emotional support providers), inefficient communication among the healthcare team, lack of communication with the VA regarding benefits such as the Community Care program, qualifications needed to apply for VA benefits, disability ratings, claims and programs available for Veterans and spouses. Also, discomfort in seeking help, inefficient communication between healthcare providers and VA Hospital, lack of communication between insurance and participant, and lack of coordination of treatments between the VA healthcare system and Community Care providers that are part of the VA network were the communication barriers identified. As a spouse shared, “*There is poor communication between the local VA Healthcare facility, where he (the Veteran) goes and the VA main Hospital in West Columbia*.” Table 3 presents the descriptive statistics of participants experiencing resources barrier or communication barriers.

**Table 3:** Descriptive statistics of participants experiencing resource barriers, communication or cultural competence barriers to access healthcare

Barriers	Frequency	Percentage
*Resource Barrier Access*		
Experiencing a resource barrier	57	46%
Did not mention a resource barrier	67	54%
**Total number of participants**	**124**	**100%**
*Type of Resource Barrier*		
Lack of prompt access to an appointment	21	36%
Financial problems	18	33%
Lack of providers	18	32%
Limited access to local clinics and hospitals	12	21%
Limited local programs available	9	16%
Problems with reimbursement	5	9%*
*Communication Barriers*		
Experiencing a communication barrier	40	32%
Did not mention a communication barrier	84	68%
**Total number of participants**	**124**	**100%**
*Types of Communication Barriers*		
Inefficient communication between participants and the healthcare team	10	21%
Inefficient communication among the healthcare team	2	4%
Lack of communication with the VA	28	58%
Not comfortable seeking help	3	6%
Lack of communication between insurance and participant	1	2%
Lack of communication/coordination of treatment between the VA and Community Care providers	3	6%*
Lack of communication/coordination of treatment between the VA and their contracted personnel	1	2%
*Cultural Competence Barriers*		
Experiencing a lack of cultural competence barrier	19	15%
Did not mention a lack of cultural competence barrier	105	85%
Total number of participants	**124**	**100%**
*Types of Lack of Cultural Competence Barrier*		
Cultural indifference (Age, Gender, Race)	6	32%
Lack of tact/knowledge on how to talk to Veterans	2	11%
Lack of knowledge on how to treat Veterans	4	21%
Lack of knowledge about Veterans	5	26%
Stigma	2	11%

Note. *The different types of resources, communication and cultural competence barriers reported do not round up to 100% since those estimates were calculated on the total number of participants who mentioned having that barrier (*n* = 57, *n* = 19, and *n* = 33, respectively). The same participant could be reporting more than one type of barrier

## Discussion

Ward et al. (2017) used a theoretical framework stressing the intersection of Veteran perceptions and actions within several contexts and sociocultural conditions [17]. According to the authors, not all Veterans are familiar with the services available at the VA and new delivery approaches, including mobile clinics or telemedicine. The lack of information about the availability of these services and technologies, the lack of understanding about the VA’s role, and the qualifications required for health and mental health services limit Veteran’ usage of these resources [17]. Veterans’ healthcare needs have changed with age. They now have to navigate a new, intricate context involving multiple types of coverage (e.g., private, VA, Medicare) and learn how to access and coordinate different sources of local or regional healthcare services [17].

The results of our study revealed that close to one-half of our Veterans participants, 44%, have dual VA and non-VA healthcare, including government healthcare programs such as Medicare or private insurance. Each insurance has its own set of rules that must be circumnavigated within the local providers and/ or throughout VA facilities. This situation can create conflicts in communication, which could cause confusion and delay Veterans’ access to needed care [17]. However, choosing non-VA healthcare providers can have a financial impact on Veterans. As a Veteran shared with us: “*It’s saddening because I didn’t have these (back) problems until I went through the military. I feel like the military should be paying for it, no matter what. They would have probably sent me to Columbia to get an MRI (4-hour back and forth). It would have been another month before I would have seen a doctor. I had been out of work with sick leave, but I don’t want to burn all my sick leave.”* Another Veteran expressed: “*When I joined the military, I was promised by the US Government if I stayed in for a career, I would have free health care for myself and my family, so for my immediate family for the rest of my life … Now, I have to pay for Medicare, and I am going to pay for Tri-Care from the government for my medical care*.”

Co-occurring with these contexts are specific characteristics of the local community, such as limited information on health care services, distance to VA health care, and local perceptions of the appropriate sources of care for Veterans to use [17]. Results of our study revealed that geographic proximity, which is the distance to the service needed, is high, and there is also a lack of public transportation or problems with transportation services.

Distance has been identified in the literature as the main barrier for rural patients to access common diagnostics services, routine specialty care, and emergency services [4]. For others, distance is a barrier to accessing VA facilities [9] or traveling long distances to urban locations to receive specialized treatments [3]. Geographic proximity, such as driving long distances or long travel time to appointments with their PCP or SCP, was a common problem for many of our participants (44%, *n* = 54). However, according to Ward et al. (2017), distance is a complex concept. It does not matter how close to a facility a patient might be if it does not offer the service needed. Also, the same distance might represent different things for different Veterans. For some, it could mean a barrier, and for others, a way of living. But, under certain conditions, it could be a burden, especially for older Veterans who require more specialty care and have more transportation barriers. Patient satisfaction may increase if more essential services are offered locally through the Veteran Health Administration (VHA) or contracted services [17].

According to the 2023 Public Transit Annual Report, most counties in SC have transportation services in at least a portion of the county, and during that year, 718 vehicles were operated, and approximately 8 million passenger trips were made [26]. However, the results of our qualitative study revealed that more than one-half (55%) of those participants reported having transportation access problems; they lack or have problems with transportation services. Also, 25% shared a need for more access to public transportation. Additionally, 20% of those reporting transportation problems said they could not drive because of age or medical conditions. This result is consistent with the literature. Buzza et al. (2011) found that distance is often a main barrier when Veterans have limited health and function or lack financial resources or when specialty and diagnostic services are required and in case of an emergency [4]. Even though the VA offers the “Veterans Transportation Services” (VTA) in SC and FL, access is limited to qualifying VA medical centers. VTA ensures that all qualifying Veterans who do not have access to their own transportation or public transportation services due to financial, medical, or other reasons can travel to qualifying VA medical facilities or authorized non-VA appointments to receive the care they need [27]. However, in certain communities, participants expressed their situation regarding VTA: *“I have [name of the transportation service provider] to provide transportation, and I missed appointments because of them.”*

Communication was another dimension identified as a barrier. Participants expressed low satisfaction with the VA healthcare team communication. They also felt more communication between the healthcare team was needed to improve care. Our findings are consistent with the literature. Gaglioti et al. studied perceptions and prevalence of comanagement between VA and non-VA primary care providers, VA and non-VA services provided to comanaged patients, and perceptions of and recommendations for communication with the VA. The authors found that participants and non-VA primary care providers were dissatisfied with the level of communication with the VA. Although the authors explained that the lack of communication does not translate to the quality of care, 42% of the Veterans agreed that poor communication with VA providers has led to poor patient outcomes [19] Miller et al. assessed Veteran’s perspectives on care coordination between the Veterans Affairs healthcare system and healthcare from the community. They also found that a lack of coordination and communication between the VA healthcare providers and community providers has led Veterans to carry the burden of coordinating their care, creating duplicated, delayed and contradictory care [20]. Which is also consistent with the results of our study. As a Veteran explained, *“The hospital and the providers haven’t filed their paperwork correctly. So, I keep getting harassed, I keep referring them to the VA through the reference number. So I feel like I’m playing medical clerk. I would tell them how to do their job and to contact the VA.”*

Despite the efforts of the VA to increase communication by sending weekly and bi-weekly emails about entitlements, programs and services available to Veterans and families [28], our participants have expressed the need to improve communication with them since they do not know what programs are available, or what they qualify for. Anecdotal comments reflect that: *“The VA needs to be able to reach out to Veterans to let them know what programs are available.”* This might be partially explained by participants’ age in our study, the majority of whom were over 65 years old (51% *n* = 64). Of those, 32% (*n* = 40) were between the ages of 65 and 74, while 19% (*n* = 24) were over the age of 75 years old.

Lack of cultural competence was another dimension identified; 33% of those experiencing this type of barrier shared examples of providers and personnel showing cultural indifference based on participants’ age, race or gender, 28% lacked knowledge about Veterans, and 22% lacked knowledge on how to treat Veterans. In comparison, 11% showed a lack of tact/knowledge on how to talk to Veterans. To interact with Veterans in a culturally competent manner, healthcare professionals and personnel must extend their knowledge to understand better how the military can shape service members, families and Veterans’ lives. They also have to tailor care delivery by considering their background, age, gender, race, ethnicity, socioeconomic status and religious values since those will affect their perceptions about health, establish expectations for care, and increase the quality of care, producing better outcomes [28]. From the resource barriers dimension, lack of prompt access to appointments, financial problems, lack of providers, lack of access to local clinics and hospitals, limited programs available, and problems with reimbursements were identified. The most common financial problems reported were high copayments and premiums, lack of adequate coverage, income ineligibility, and gas expenses incurred by traveling long distances to access care. Goins et al. (2005) found that the cost of care and prescription medications were consistent barriers for older adults [3]. According to public law 110–387, the Veterans’ Mental Health and Other Care Improvements Act of 2008, the Veterans Health Administration (VHA) covers certain emergency services within specific parameters. However, patients, VA staff, and providers may be confused about what procedures to follow, resulting in unnecessary expenses for the Veteran. Anecdotal comments from participants support those issues: *“It was not very clear-cut about the procedures when he (the Veteran) needed emergency care, and he tried to follow every single step along the way. We followed every single step through the process. The Hospital tried to charge him, even though he talked to the VA on the phone, talked to the doctor’s office about what else he might need to do not to get charged.”* This result is consistent with results from Buzza et al. (2011) described in their findings the belief by Veterans that they must travel to a VA medical center or risk incurring significant medical costs because of confusion about what procedures to follow. The authors concluded that this may create a false barrier resulting in delay or avoidance of care [4]. Lack of prompt access to appointments was a common issue reported by Veterans who reported delays in scheduling appointments and slow clinic responses to appointments. Staff issues such as lack of providers (32%, *n*=18) were also identified as barriers. Cheney et al. (2018) found similar results supporting the current study’s finding that challenges to scheduling appointments and lack of providers created barriers to timely and appropriate care [9]. Problems with transportation reimbursement were also identified as a barrier. Participants proposed that by establishing partnerships in the communities, the VA can save money used for paying reimbursement: *“The VA can partner up and use the labs, the X-rays, and whatever is right here in the community instead of turning in a voucher for reimbursement for mileage. There are 123 Veterans, roughly, in the city of Arcadia. There are 2,200 in the county, you can do the math.”*

The Veterans Access, Choice, And Accountability Act Of 2014 aimed to improve access to quality healthcare for Veterans enrolled in the VA healthcare system and to expand the VA’s capacity to provide care to Veterans in a timely fashion [29]. Veterans can access healthcare beyond VA providers under the Community Care Program, where the VA pays for care from local community providers for qualifying Veterans when they cannot provide the service needed [30]. Even though this program has been available to Veterans that qualify for the VA Healthcare system, the results of our study revealed that most participants (68%, *n* = 84) are unaware of such a program.

## Conclusions

Our study reinforces the value of collecting a deeper understanding of the dimensions to access to mental and physical healthcare that rural Veterans and their families are experiencing. We identified five dimensions of access to healthcare:

1. Geographic proximity, which includes travel time and distance to the service needed (e.g., primary care and specialty care providers);
2. Transportation problems, such as lack of personal transportation, public transport and or problems with transportation services;
3. Resource issues include lack of prompt access to appointments, financial problems, lack providers, limited access to local clinics, limited local programs available and problems with reimbursement;
4. Communication issues, including a lack of communication with the VA and inefficient communication between the healthcare team and between the Veteran and the healthcare team and lack of communication/ coordination of care between the VA and the Community Care service providers, and.
5. Lack of cultural competencies, including cultural indifference based on Veteran’s age, race, and gender, and lack of knowledge about Veterans and how to treat and talk to them.

Partnering with local community providers will reduce some of the burdens Veterans incur. Veterans can save time, reduce travel costs and remove the inconvenience of applying for reimbursement, particularly for those who are not technologically savvy. Our findings suggest that, while resources such as the VA “Community Care Program” are available to Veterans in rural communities, improving communication between Veterans and the VA healthcare system could increase perception of availability and increase trust and satisfaction with the VA health system. Additionally, ensuring healthcare providers and contracted personnel are culturally competent to work with Veterans might improve communication barriers, trust, and adherence to treatment. Ameliorating communication issues between primary care providers and specialty care providers, and among the healthcare team, and healthcare providers with Veterans, and improving coordination of care between the VA health system and Community providers could lead to improved patient health outcomes.

### Study strengths and limitations

Veteran and family of Veteran data are critical for VA and non-VA healthcare policymakers. One strength of this qualitative study, therefore, is that findings are directly applicable to the Veteran population since the demographic characteristics of our sampled population of Veterans share similarities with Veterans residing in SC and FL and the total Veteran population in the United States. Barriers identified in this study can be addressed to directly impact rural Veterans’ access to healthcare. The structured interviews used in this study also provided insight into Veterans’ current knowledge and usage of the VA Healthcare system, usage of dual VA and non-VA healthcare services, or usage of non-VA healthcare such as Medicare or other private insurance by Veterans and spouses. In-depth interviews revealed a specific program offered by the VA called the “Community Care Program.” After uncovering the program from a small number of initial interviews, researchers were able to prompt Veterans with questions about the specific program in subsequent interviews. Change in the interview question; however, also presents a limitation to the study. Despite efforts to ensure the accuracy of participants’ responses about using the VA Community Care Program versus general care from community resources, potential for errors in interview coding exists.

Even though we addressed that participants experienced barriers to accessing healthcare, specifically to appointments with their primary or specialty care provider, we did not include the frequency and percentages of participants’ type of visit, if it was preventative care with a new or own provider, if they had an appointment with a specialty care provider, if it was urgent care or emergency care. Many of our participants specified that there was no Hospital in their county; others referred only to generalities about how far the VA Hospital or clinic was from where they lived and how much they had to drive. We did not include information about visits to urgent care and if they were ambulatory or had to go to the nearest medical center. These types of visits could have been impacted by transportation issues or geographic distance to care.

Finally, we did not include information about the community physician’s offices/medical practices that are accessible and available. Many participants disclosed that they did not know about the VA Community Care program, and many had heard about it but never used it.

### Implications for the veteran health administration (VHA)

The results of our study revealed that Veterans and their families are not receiving the healthcare access that they need for good health. High geographic proximity (distance or travel time) to the needed service provider, lack of communication about programs and services availability, limited access to local clinics and programs, lack of transportation or problems with transportation services, lack of prompt access to appointments, and lack of providers were some of the barriers encountered in rural regions of SC and FL. Improving awareness of programs available, such as the “Community Care Program,” is needed. Promoting partnerships with existing transportation services is needed in locations lacking public transportation. Improving waiting time by partnering with local providers or raising awareness about the Community Care Program will improve healthcare utilization, health outcomes, and patient satisfaction. Improving communication between the Veterans and their families and the local VA offices is needed. Some Veterans said they were not technology savvy or had no active email accounts. Therefore, it would be vital for them to receive information about programs and resources available through a print newsletter.

### Participants suggestions to the VHA to improve access to healthcare

Having mobile health clinics or satellite offices that offer screening and/or mental health services in rural areas will help with the lack of specialized providers and the distance to VA hospitals or clinics. Additionally, making telehealth or videoconference medical services available to Veterans in rural areas can reduce wait time for appointments and the number of hours full-time employed Veterans take out of work for medical appointments. To help with the transition and reintegration into family life and to help find and maintain a job, it is essential to have support group programs available for Veterans and spouses in rural areas. Having transportation services available in rural areas, especially those without public transportation, is vital. Participants also suggested having kiosks available at the VA office to ease the process of transportation reimbursement. To improve communication with the VA, participants proposed in-person meetings/seminars at the local VA office to learn what programs/benefits are available and inform what is available before leaving active duty. They also suggested that the VA advertise in the newspaper or radio. Participants asked for resource navigation to help them find what they were looking for, such as whom to contact for information about benefits, claims, financial assistance, support programs for Veterans, spouses and families, support caregiver programs, etc. It is also important to highlight that participants expressed the need for more culturally competent healthcare providers and contracted personnel who listen to Veteran patients’ needs.

### Implications for future research

While increasing efforts to bring awareness to existing resources for accessing healthcare services is needed, future research should focus on identifying additional pathways to meet the needs of rural Veterans.

### Electronic supplementary material

Below is the link to the electronic supplementary material.

Supplementary Material 1

## Data Availability

The datasets used and/or analyzed during the current study are available from the corresponding author upon reasonable request.
