# Supplementary material for: Rural veteran perception of healthcare access in South Carolina and Florida: a qualitative study
Source: BMC Health Serv Res. 2024 Jul 20;24:826. doi: 10.1186/s12913-024-11241-3 (PMC11264868; doi:10.1186/s12913-024-11241-3)
Supplement: Supplementary file 1 — Supplementary Material 1 [file 12913_2024_11241_MOESM1_ESM.doc]

**Rural Veterans and Spouses/Partners: Individual Interview**

Thank you for being a part of this important study. We are interested in understanding the health needs and experiences of veterans and their families in rural South Carolina/Florida to help the Veterans Affairs (VA) and the Cooperative Extension Service in SC and FL better serve your needs. First, I would like to ask you a few questions about you and others living in your home. For each of the following questions, please choose the answer that best describes your situation. There is no right or wrong answer. All the information you offer me will be anonymous and strictly confidential. With your permission, I will be recording our conversation, but will not refer to you by name or other ask other identifying information. We will destroy the recording as soon as we have written down your words. In addition to never using your real name, we promise that we will take extra steps to make it harder for people to identify you. All your responses will only be used for academic purposes. Thank you for your participation!

**I. Preliminary Screening Questions**

1. What is your current zip code?
2. What is your current address?
3. Where/how you were recruited into the military?
4. What was your rank when you left the military?

If the individual is unable to answer one or more of the above questions OR is unable to answer the questions in a reasonable amount of time, ask:

1. What is the capital of South Carolina?
2. Who is the governor of South Carolina?
3. What states border South Carolina?
4. What is the closest VA office to you?

**II. Getting to Know You: Demographic Characteristics**

**[INTERVIEWER: Prior to the interview, transfer the following information from the screener to the protocol.]**

**When we spoke with you about your interest in the veteran health care study, you indicated that:**

1. **You are a veteran OR the spouse/partner of a veteran.**

**Is that correct?**

**Great. Now can you tell me…**

1. **Your age**
   1. 18-29
   2. 30-39
   3. 40-49
   4. 50-64
   5. 65-74
   6. 75 and older
2. **What is your gender?**
3. **Your household family members are yourself and (any spouse and/or children) ___**
4. **Your approximate household income is**
   1. $24,999 or below
   2. $25,000 to $49,000
   3. $45,000 to $74,999
   4. $75,000 to $99,999
   5. $100,000 and above
5. **And you identify your race/ethnicity as** ____

**7. What is your current marital status? *(Check one.)***

____ Single/Never Married

____ Married

____ Partnered, unmarried

____ Separated

____ Divorced

____ Widowed

**8.  What is your current employment status?**

____ Working full-time

____ Working part-time

____ Retired

____Not employed

**9a.** *[If veteran:]* **In which branch of the armed services did you serve?**

**9b. In what years did you serve in [service branch]?**

**9c. Did you experience combat?**

**10a.** *[If not veteran:]* **In what branch of the armed services did your spouse/partner serve? __________________**

**10b. In what years did your partner serve in [service branch]? _______________**

**10c. Did your partner experience combat? _______________**

**III. Needs Assessment**

**Now, I am going to ask you a few questions about your health and your experience with the healthcare system.**

**11**. **How is your health?**

Or: How would you rate your health on a scale of 1 to 10, with 1 being extremely poor and 10 being in tip-top shape?

Prompts if it doesn't come up:

- - What health issues do you have?
  - What do you do to maintain your health?
  - Are you able to get the care you need? If not, what gets in the way?
  - Do you have any healthcare coverage? If so, what kind and how has that worked for you?
  - Do you ever used the VA or veteran-specific health resources? How have those worked?
  - Have you ever used Community Care? If so, what has been your experience?

**12. We’ve talked about your physical health, but what is your mental health like?**

Prompts if it doesn't come up:

- How much and what kinds of stress are you experiencing? What makes you worry or upset?
- What mental health issues do you have?
- What do you do to maintain your mental health?
- Are you able to get the care you need? If not, what gets in the way?

**13.** *(If the respondent has children)* **How is your children’s health?**

Prompts if it doesn't come up:

- - What health issues do they have?
  - What do you do to help maintain their health?
  - Are they able to get the care you need? If not, what gets in the way?
  - Do your children have healthcare coverage? If so, what kind and how has that worked for them?
  - Do you ever use the VA or veteran-specific health resources? How have those worked?

**14. What is your children’s mental health like?**

Prompts if it doesn't come up:

- How much and what kinds of stress are your children experiencing? What makes them worry or upset?
- What mental health issues do they have?
- What is done to maintain their mental health?
- Are they able to get the care they need? If not, what gets in the way?

**III. Extension**

**15. What do you know about Cooperative Extension?**

- - Have you ever worked for Extension or attended any programs? What has your interaction been with Extension?
  - If you have worked with Extension, what programs did you attend, and were they helpful? What is your general impression of Extension?

**16. Lastly, is there anything else you would like to say that would help us better understand the issues facing rural veterans?**

***Thank you for taking the time to complete this interview!***
